# Comparative Evaluation of ( ^18^ F)AlF-PSMA-HBED-CC and ^68^ Ga-PSMA-HBED-CC in Staging Intermediate-/High-Risk Prostate Cancer: A Prospective Study

**DOI:** 10.1055/s-0045-1801842

**Published:** 2025-01-21

**Authors:** Gerardo Gabriel dos Santos Loureiro, Pablo Duarte Couto, Juan Pablo Gambini Gonzalez, Omar Alonso Nuñez

**Affiliations:** 1 Uruguayan Centre of Molecular Imaging (CUDIM), Montevideo, Uruguay; 2 Nuclear Medicine and Molecular Imaging Centre, Hospital de Clínicas, Universidad de la República, Montevideo, Uruguay

**Keywords:** high-risk prostate cancer, initial staging, ^18^ F-AlF-PSMA-11, ^68^ Ga-PSMA-11, PET/CT

## Abstract

**Objective** ^68^ Ga-PSMA-HBED-CC positron emission tomography (PET)/computed tomography (CT) represents a clinically relevant technique for the evaluation of prostate cancer (PCa) patients, whereas ^18^ F-AIF-PSMA-HBED-CC is a novel tracer produced in our center, with suitable radiochemical purity for clinical purposes. We prospectively compared the diagnostic values of both tracers for the detection of metastatic disease in patients with intermediate-/high-risk PCa at initial staging.

**Materials and Methods** Sixty-six patients (mean age: 63 years; range: 52–78 years) with PCa at initial staging (Gleason score ≥6; median prostate-specific antigen [PSA]: 10 ng/mL; range:1.7–152 ng/mL) prospectively underwent routine ^68^ Ga-PSMA-11 and ^18^ F-AlF-PSMA-11 PET/CT scanning with a 64-slice PET/CT scan with time-of-flight (TOF) correction. We measured the maximum standardized uptake value (SUVmax) and lesion-to-background ratio (LBR) in all coincidentally detected lesions. Open prostatectomy and pelvic lymph node dissection were performed in nonmetastatic patients. Histopathology, correlative imaging, and/or clinical follow-up were considered the gold standard. Follow-up was conducted at least 4 months after PET/CT scanning (median: 6.4 months; range: 4–11 months). Sensitivity, specificity, and predictive values were calculated.

**Results** The overall detection rate was 85% (56/66) for both tracers. At least one suspicious lesion indicating potential PCa metastasis was detected in 20 (30%) and 21 (32%) of 66 patients for ^68^ Ga-PSMA-11 and ^18^ F-AIF-PSMA-11 tracers, respectively. A total of 145 extra-prostatic lesions were detected in the bone ( *n* = 56), lymph nodes ( *n* = 88), and lung ( *n* = 1) by at least one radiopharmaceutical: 131 (90%) for ^68^ Ga-PSMA-11 and 123 (85%) for ^18^ F-AlF-PSMA-11.

In concordant lesions, a significant correlation was found between the SUVmax of both tracers ( *r* = 0.90, *p* = 0.001). The SUVmax and LBR for ^18^ F-AlF-PSMA-11 were higher in bone foci ( *n* = 39) compared with ^68^ Ga-PSMA-11 (7.2 vs. 8.9 and 14 vs. 13, respectively, *p* = 0.02).

For the detection of systemic metastasis, the sensitivity values were the same for both techniques: 0.90 (95% confidence interval [CI]: 0.68–0.98). We calculated specificities of 0.96 (95% CI: 0.85–0.99) and 0.94 (95% CI: 0.82–0.98) for ^68^ Ga-PSMA-11 and ^18^ F-AlF-PSMA-11, respectively.

**Conclusions** ^68^ Ga-PSMA-11 and ^18^ F-AlF-PSMA-11 PET/CT seem to be clinically equivalent imaging techniques for the assessment of primary intermediate-/high-risk PCa with promising potential for the detection of metastatic spread that would impact patient management.

## Introduction

Prostate cancer (PCa) is the second most common cancer in men, with a high prevalence and incidence worldwide. 1

Optimal treatment in high-grade tumors with a Gleason score ≥7 includes purportedly curative prostatectomy and/or radiotherapy. A cancer-specific survival rate of 85% at 10 years has been demonstrated. 2 However, at least 12% of patients have metastatic involvement and consequently a shorter survival time. 3

The well-known low sensitivity at low prostate-specific antigen (PSA) levels of the previous gold standard radiolabeled choline led to the search for new tracers with better performance. 4

Prostate-specific membrane antigen (PSMA) targeted positron emission tomography (PET)/computed tomography (CT) has proven to be a highly accurate method of detecting PCa lesions, with better diagnostic accuracy than that of choline. PSMA overexpression on the cell surface of PCa cells, especially at low PSA levels, is well documented. 5

The ^68^ Ga-labeled PSMA-targeted PET/CT tracer Glu-urea-Lys(Ahx)-HBED-CC (PSMA-11) has proven to be highly sensitive in the detection of recurrent PCa, 6 but it has some limitations, such as a short half-life and limited activity per synthesis. 7

Several ^18^ F-labeled analogs were introduced into clinical PET imaging in recent years, which present many advantages over gallium: production on large scale, reasonable cost due to cyclotron ^18^ F production, lower positron energy with higher image quality, and a more practical half-life that allows for the acquisition of delayed images. The most studied ^18^ F-labeled tracers are ^18^ F-DCFBC, ^18^ F-DCFPyL, and, most recently, ^18^ F-PSMA-1007. 8 9

A novel agent, ^18^ F-AlF-PSMA-11, was produced at our center with suitable radiochemical purity for clinical purposes 10 and with the additional advantage of being cheap, since the precursors are synthesized in situ.

Most data outlining the utility of PSMA-PET techniques came from the setting of biochemical recurrence after definitive therapy.

The purpose of this study was to prospectively compare the diagnostic performance of ^18^ F-AlF-PSMA-11 and ^68^ Ga-PSMA-11 in the detection of metastasis in patients with intermediate-/high-risk PCa at initial staging prior to therapy and their potential impact on patient management.

## Materials and Methods

Between September 2017 and March 2019, we prospectively analyzed 66 patients (mean age: 63 years; range: 52–78; median PSA: 26.5 ng/mL; range: 1.7–152 ng/mL; Table 1 ) with localized intermediate-/high-risk PCa according to the D'Amico classification, 11 prior to radical prostatectomy (Gleason score ≥6). Patients with confirmed metastasis, concomitant cancers, or benign pathology were excluded. Patients underwent clinical and laboratory preoperative staging with PSA, biopsy Gleason score grading, bone scintigraphy, and CT.

**Table 1:** Patients' characteristics

Patient's characteristics	
Age (y)	Mean: 63.1 ± 6.5; median: 63; range 52–78
PSA (ng/mL)	Mean: 26.5 ± 31.4; median: 14; range 1.7–152
Gleasson score of 6	8 patients (12.1%)
Gleasson score of 7	38 patients (57.6%)
Gleasson score of 8	11 patients (16.6%)
Gleasson score of 9	8 patients (12.2%)
Gleasson score of 10	1 patient (1.5%)

Abbreviation: PSA, prostate-specific antigen.

### Radiopharmaceuticals

#### ^18^ F-AlF-PSMA-11

The complex ( ^18^ F)AlF-PSMA was obtained from an automated Tracerlab FXFN (GE) platform. A radiochemical purity higher than 90% (95 ± 3%) was achieved. Stability was verified for 4 hours in the final formulation vial and for up to 1 hour in human plasma. 10

#### ^68^ Ga-PSMA-11

^68^ Ga-PSMA-11 was produced using PSMA-11 (HBED-CC) from ABX as a precursor and ^68^ Ga eluted from an ^68^ Ge/ ^68^ Ga generator (ITG, Germany). The precursor (3.2–3.6 nmol) was dissolved in ultrapure water and mixed with 1.00 mL of 0.25-M sodium acetate and 650- to 1,450-MBq ^68^ GaCl _3_ in 4 mL of 0.05-M HCl. After 5 minutes of incubation at 100°C, ^68^ Ga-PSMA-11 was purified and sterilized. The radiochemical purity was 99.2 ± 1.7% and specific activity was 170 ± 76 MBq/nmol.

### Imaging

All patients underwent a 64-slice PET/CT scan with both ^18^ F-AlF-PSMA-11 and ^68^ Ga-PSMA-11 within 1 to 2 weeks. The scans were performed 60 minutes after tracer injection, with a dose of 4.0 MBq/kg for ^18^ F-AlF-PSMA-11 and 2.0 MBq/kg for ^68^ Ga-PSMA-11.

The CT parameters were the following: tube voltage, 120 kVp; autoMA, 80 to 180 mA; index noise, 30, “GE SmartMa dose modulation”; rotation time, 0.8 seconds; rotation length/full helical thickness, 3.75 mm; pitch, 1.375:1; and speed, 55 mm/rotation. PET data were acquired in 3D with a scan duration of 2 minutes ( ^18^ F-AlF-PSMA-11) or 3 minutes ( ^68^ Ga-PSMA-11) per bed position and with 11-slice overlap. Images were reconstructed using an ordered subset expectation maximization algorithm (OSEM) with time-of-flight correction (matrix size: 128 × 128 pixels) with 2 iterations/24 subsets.

### Image Analysis

Images were evaluated by two board-certified specialists in nuclear medicine and by one radiologist.

Suspicious metastatic lesions (“positive” study) were defined as any focal uptake at any location, higher than surrounding background or normal tissue, localized by hybrid images, excluding joint processes and areas of physiological uptake. 12 For image interpretation, we also consider the Promise criteria, the recently published E-PSMA (European Association of Nuclear Medicine-EANM standardized reporting guidelines por PSMA-PET imaging in prostate cancer) and the PSMA-RADS (Prostate-specific Membrane Antigen Reporting and Data System). 13 14

Lesions were evaluated regarding their localization (bone, lymph node [LN], or soft tissue metastases) and their maximum standardized uptake values (SUVmax) selecting gluteal musculature as background.

We measured the lesion-to-background ratio (lesion-SUVmax/background-SUVmax; LBR) in all prostate glands and in all coincident lesions. Afterward, prostatectomy and pelvic LN dissection were performed in nonmetastatic patients.

### Gold Standard

Histopathology, correlative imaging, and/or clinical follow-up were considered the reference standard to assess the diagnostic performance on a per-patient and per-lesion basis as follows:

- Lesions that were visually considered as suggestive of PCa:
  – Histological confirmation.
  – Areas of abnormally increased tracer uptake (multifocal metastatic disease).
  – A metastatic lesion on an imaging modality other than the one performed as the baseline PET scan, either in the reevaluation of previous studies or in subsequent examinations.
  – Increase in the number or size of bone or soft tissue lesion(s) from baseline PET scan during follow-up.
  – Decrease in the number or size of bone or soft tissue lesion(s) following disease-specific treatment.
  – Presence of a lesion on baseline scans in a patient with symptoms suggesting malignancy.
  – Increasing or decreasing PSA levels in agreement with a clinical scenario of progression/response.
  – Unequivocal positive findings at baseline PET scan that persisted during follow-up in a setting of PSA greater than 0.2 ng/mL, at least 3 weeks following prostatectomy.

### Statistical Analysis

A systematic comparison was performed between the results obtained with both tracers regarding the number of detected PET-positive lesions, the SUVmax value, the LBR, the PSA, and Gleason score. The assessment of the normality distribution of variables was done via the Shapiro–Wilk test. The nonparametric Spearman rho test and the Pearson correlation coefficient were used to measure the strength of association between SUVmax and LBR in all concordant lesions. The tumor SUVmax and tumor-to-background ratio signals from the same lesions in both the ^18^ F-AlF-PSMA-11 and ^68^ Ga-PSMA-11 studies were statistically analyzed using a nonparametric two-sided Wilcoxon signed-rank test and the Student paired *t* -test. The two-sided McNemar test was used to analyze whether ^68^ Ga-PSMA-11 PET/CT detects significantly more lesions suggestive of PCa when compared with AlF-based PET/CT. Sensitivity and specificity were selected to describe the diagnostic performance of PET/CT in detecting metastasis involvement. A *p* -value of 0.05 was considered significant. For statistical analyses, we used the Statistical Package for the Social Sciences (SPSS) version 23.

## Results

We enrolled a total of 66 patients. The mean age was 63.1 ± 6.5 years (median: 63 years; range: 52–78 years) and the mean PSA level was 26.5 ± 31.4 ng/mL (median: 14 ng/mL; range: 1.7–152 ng/mL).

Both PET tracers demonstrated abnormal findings in 56 patients (positivity rate:85%) and were negative in 10 patients (15%). At least one lesion suspicious for metastasis was detected in 20/66 (30%) and 21/66 (32%) patients for ^68^ Ga-PSMA-11 and ^18^ F-AlF-PSMA-11 PET/CT, respectively ( Table 2 ).

Prostatic focal lesions that showed moderate to high radiotracer uptake above the SUVmax of 2.5 were seen in 54/66 patients (81.8%) in ^68^ Ga-PSMA-11 PET/CT scans and in 53/66 patients (80.3%) in ^18^ F-AlF-PSMA-11 PET/CT scans. In the remaining patients, the primary tumor showed no tracer accumulation or only diffuse tracer accumulation.

**Fig. 1:**
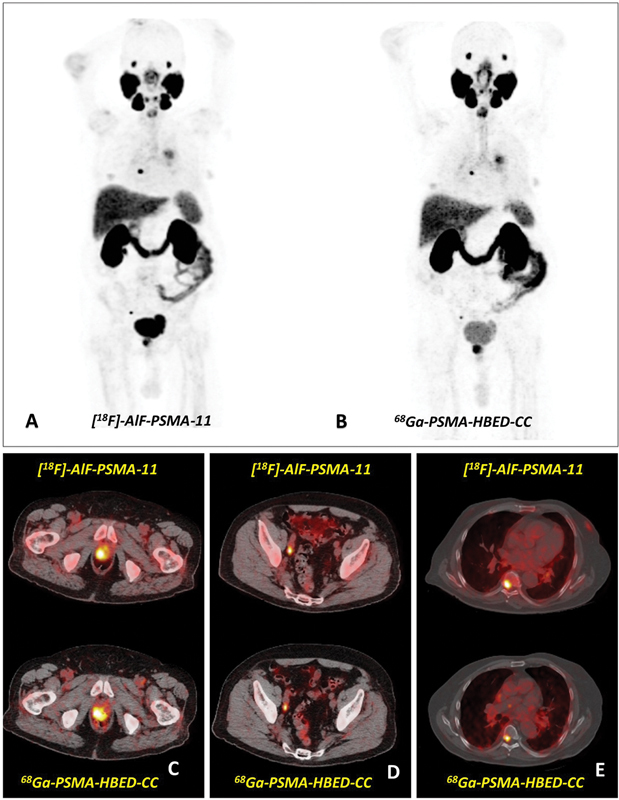
Maximum intensity projection (MIP) and axial slices of fusion positron emission tomography (PET)/computed tomography (CT) of a patient with early-stage prostate cancer scanned with ( **A** ) ^18^ F-AlF-PSMA-11 and ( **B** ) ^68^ Ga-PSMA-11 PET/CT. ( **C** ) Prostate lesion, ( **D** ) right external iliac lymph node metastasis, and ( **E** ) bone lesion in T7 demonstrated with both tracers.

**Fig. 2:**
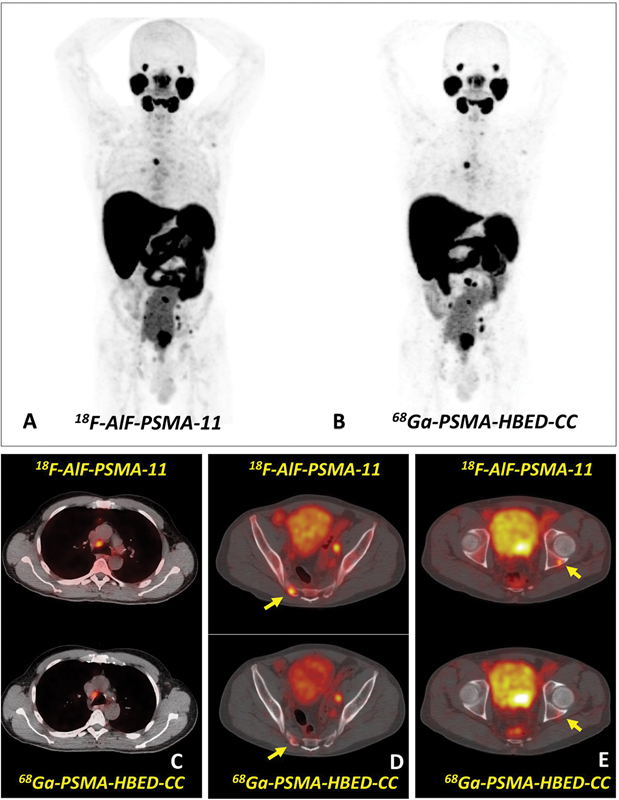
Maximum intensity projection (MIP) of ^18^ F- AlF-PSMA-11 ( **A** ) and ^68^ Ga-PSMA-11 ( **B** ) and axial slices of fusion positron emission tomography (PET)/computed tomography (CT) demonstrating coincident metastatic lymph node and bone lesions ( **C –E** ). Note the better contrast and uptake intensity of ^18^ F-AlF-PSMA images in some bone lesions (yellow arrows).

A total of 145 extra-prostatic lesions were detected by at least one radiopharmaceutical in bone ( *n* = 56), LNs ( *n* = 88), and lung ( *n* = 1), for a total of 131 for ^68^ Ga-PSMA-11 and 123 for ^18^ F-AlF-PSMA-11. Discriminating by radiotracer in the total population, PET/CT with gallium and fluorine identified 42 and 53 bone lesions and 88 and 69 LN lesions, respectively ( Figs. 1 and 2 ).

In concordant lesions ( *n* = 177), a significant correlation was found between the SUVmax of both radiopharmaceuticals ( *r* = 0.90; 95% confidence interval [CI]: 0.87–0.93; *p* = 0.001). The correlation between the ^68^ Ga-PSMA-11 and ^18^ F-AlF-PSMA-11 SUVmax ratio and background lesions was also significant (0.91; 95% CI: 0.89–0.94; *p* = 0.001). The global median SUVmax showed higher values for ^68^ Ga-PSMA-11 (9.1 ± 9.6 vs. 7 ± 9.5; r=0.91); also, the median was higher for ^68^ Ga-PSMA-11 LBR (13.3 ± 23.6 vs. 12.0 ± 16.7; *p* = 0.001 for both tests).

**Fig. 3:**
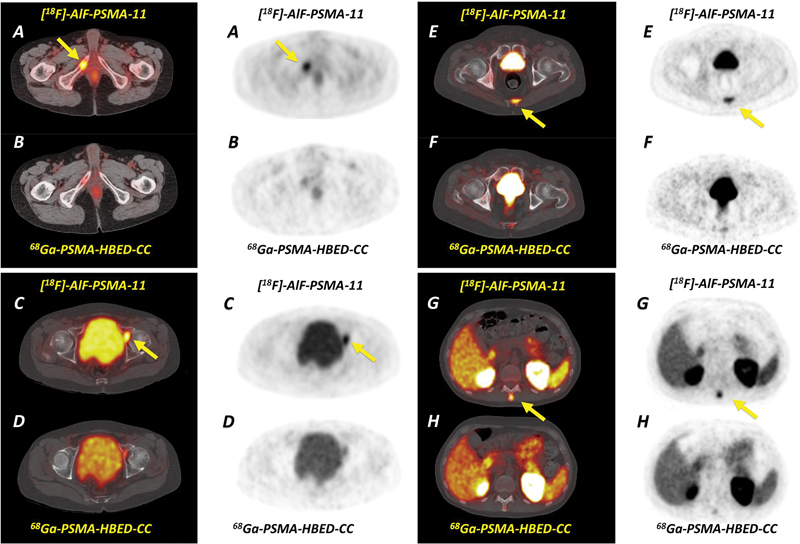
^18^ F-AlF-PSMA-11 and ^68^ Ga-PSMA-11 positron emission tomography (PET)/computed tomography (CT) images show abnormal tracer uptake in unsuspected metastases in the right ischiopubic ramus ( **A - B** ), left acetabulum ( **C-D** ), coccyx ( **E-F** ), and in the spinous process of T12 ( **G-H** ). Note the poor bone uptake with ^68^ Ga-PSMA-11.

We found a significantly higher median SUVmax for ^68^ Ga-PSMA-11 compared with that of ^18^ F-AlF-PSMA-11 in LN ( *n* = 69) and prostate foci ( *n* = 69): 10.2 ± 12.5 (3.00–74.12) versus 7.07 ± 12.8 (2.91–72.92) and 7.86 ± 7.2 (2.79–34.93) versus 7.4 ± 6.3 (2.00–34.93) for each tracer, respectively ( *p* = 0.001). The ^18^ F-AlF-PSMA-11 SUVmax and LBR were higher in bone foci ( *n* = 39) compared with that of ^68^ Ga-PSMA-11 (8.92 ± 12.8 vs. 7.2 ± 12.5 and 13.0 ± 8.6 vs. 10.9 ± 8.9, respectively; *p* = 0.02; Fig. 3 ).

Rising SUVmax values were not associated with rising Gleason categories in ^68^ Ga-PSMA-11 ( *r* = 0.01; *p* = 0.88) or ^18^ F-AlF-PSMA-11 PET/CT ( *r* = 0.02; *p* = 0.753). No significant associations were observed between PSA and SUVmax gallium (Spearman's rho = 0.001; *p* = 0.986) or fluorine (Spearman's rho = 0.089; *p* = 0.188). No relationship was found between the SUVmax and the location of the detected abnormal foci.

There was a strong correlation between the number of lesions detected by gallium and by fluorine ( *r* = 0.96; Spearman's rho = 0.95; *p* = 0.001). We also found a significant correlation between the Gleason score and the number of lesions detected by both fluorine and gallium ( *r* = 0.214 and 0.215; Spearman's rho = 0.31 for each tracer; *p* = 0.01).

The correlations between PSA and the number of lesions detected were weakly positive ( *r* = 0.355 with gallium and *r* = 0.25 with fluorine; Spearman's rho = 0.24 and 0.25, respectively; *p* = 0.06 and 0.04, respectively).

We studied the diagnostic accuracy of both techniques in identifying regional and distant metastases. Of the total patient sample ( *n* = 66), 28 underwent radical prostatectomy with extended pelvic lymphadenectomy. The rest of the patients were treated with radiation therapy ( *n* = 38; 57.6%), associated or not associated with hormone therapy.

For the detection of systemic metastasis on a per-patient basis, the sensitivity values, with their 95% CI, were the same for both techniques: 0.90 (95% CI: 0.68–0.98). We calculated specificities of 0.96 (95% CI: 0.85–0.99) and 0.94 (95% CI: 0.82–0.98) for ^68^ Ga-PSMA-11 and ^18^ F-AlF-PSMA-11, respectively.

In a per-lesion analysis, the total number of lesions studied was 225, of which 219 were gold standard positive. There were six false-positive bone results in three patients detected with both tracers, corresponding to lesions with moderate diffused uptake.

PET/CT changed clinical management in 45/66 patients (68%) who were upstaged due to the detection of bone lesions or extra-pelvic LNs that were undetectable by other modalities (bone scan, technetium imaging, or magnetic resonance [MR] imaging). Treatment was thus shifted from surgery or local radiation to systemic therapy in those patients.

## Discussion

Our preliminary data highlight the growing body of evidence supporting the use of radiolabeled PSMA-PET/CT for primary staging of intermediate-/high-risk PCa patients.

The introduction of PET/CT and PET/MR technology based on molecular information has led to a revolution in imaging diagnosis in PCa, improving the detection of metastatic disease compared with CT or MRI. 15

Radioactive labeled choline showed a high specificity (95%) but only a moderate sensitivity (33–50%) in the detection of metastatic LN spread, 16 and resulted in research for more sensitive radiotracers. 17

The glutamate carboxypeptidase type 2 PSMA is overexpressed on the cell surface of PCa cells specially in advanced-stage and castration-resistant metastatic PCa. 18 The most widely studied agent based on low-molecular-weight urea type structures is the ^68^ Ga-labeled PSMA inhibitor Glu-NH-CO-NH-Lys(Ahx)-HBED-CC. In addition, after this tracer binds to PSMA, internalization occurs, and this can be used for a theragnostic approach using ^177^ lutetium. 19

Several studies have demonstrated the diagnostic superiority of ^68^ Ga-PSMA versus choline PET/CT in assessing PCa patients. 12 17 18 This was also reported by our group. 20 However, ^68^ Ga-labeled compounds are produced with generators providing limited activity per synthesis (1–4 patients per batch) and have some shortcomings, such as a short half-life (68 minutes) and nonideal energies. 7

Compared with ^68^ Ga-labeling compounds, ^18^ F-PSMA agents constitute an attractive alternative that offers promising advantages concerning availability, production amount, and image resolution. 21 Dietlein et al demonstrate the advantage of labeling this type of peptide with ^18^ F, with the additional advantage of transportation from in situ cyclotron to distant PET centers. 7

Mease et al described a first generation of ^18^ F-labeled PSMA ligand, ^18^ F-DCFBC, with the potential for the detection of metastatic PCa. 8 Chen et al have introduced a second generation of ^18^ F-labeled PSMA ligand, ^18^ F-DCFPyL, suggesting its high potential for PSMA radiolabeled PET imaging. 22 Recently, it has been demonstrated that ^18^ F-PSMA-1007 presents similar behavior to that of ^68^ Ga-PSMA-11 and other ^18^ F-labeled PET tracers, with reduced urinary clearance. 9

In addition, recent research has emerged using Al ^18^ F labeling in patients. Yu et al 23 performed an evaluation of integrins labeled with Al ^18^ F in healthy volunteers. In recent years, the possibility of labeling PSMA with ^18^ F has emerged using the AlF + complex.

Based on the work of Allott et al, 24 tagging and purification of ^18^ F-AlF-PSMA was optimized, transferring the manual synthesis to an automatic module and producing a batch of the radiopharmaceutical with high activity in a safe and effective way.

Our group was the first to optimize the synthesis of an Al ^18^ F radiofluorinated GLU-UREA-LYS(AHX)-HBED-CC PSMA ligand in an automated synthesis platform with suitable radiochemical purity for clinical purposes expecting that ^18^ F-AlF-PSMA-11 would perform comparably to other ^18^ F-labeled PSMA targeted tracers, with the additional advantage of low cost. 10

Recently, ^18^ F-AlF-PSMA-11 has been suggested as a novel and attractive alternative to ^68^ Ga-PSMA-11 and other ^18^ F-PSMA tracers with accessibility and commercial advantages as well as similar tumor uptake among them. 25 26

Interestingly, a recent study made a head-to-head comparison between ^18^ F-AlF-PSMA-11 and ^68^ Ga-PSMA-11 in diagnosing PCa. They demonstrated that ^18^ F-AlF-PSMA-11 can bind to receptors stably and persistently; in the meantime, a clearer metabolic background was manifested when compared with ^68^ Ga-PSMA-11 PET, indicating that ^18^ F-AlF-PSMA-11 has great potential as an alternative PSMA imaging agent to ^68^ Ga-PSMA-11 since it allows for further characterization of lesions through delayed imaging. 27

Finally, while many studies have been published on the role of radiolabeled PSMA-PET/CT in the recurrent PCa biochemical scenario, only a small number of studies explored its use in the primary staging of patients with intermediate-/high-risk PCa prior to therapy. 28 Notably, radiolabeled PSMA-PET imaging in combination with multiparametric MRI could enable a complete staging with increased accuracy and additional molecular information. 29

In this prospective, descriptive, and observational study, we compared the diagnostic value of ^68^ Ga-PSMA-11 PET/CT with that of ^18^ F-AlF-PSMA-11 for the detection of metastatic disease in 66 patients with intermediate-/high-risk PCa at initial staging prior to radical prostatectomy and their overall impact on patients' management plans.

Each lesion was delineated as a true- or false-positive/negative result based on follow-up, imaging, biopsy, and follow-up PSA levels after treatment. 30 Lesions indicative of PCa in PET/CT were detected in 56/66 patients (85%) with both tracers.

Abnormal prostate gland foci were clearly observed with ^68^ Ga-PSMA-11 ( *n* = 54; 81.8%) or ^18^ F-AlF-PSMA-11 ( *n* = 53; 80.3%), while the rest of the patients showed non/diffuse/irregular uptake. These data are similar to previous reports in which it was revealed that more than 90% of primary PCa show moderate to high PSMA expression levels by ^68^ Ga-PSMA-PET. 31 It is known that less than 10% of primary cancer tumors may not overexpress PSMA 28 and that false-negative scans may occur if PCa is poorly differentiated or displays neuroendocrine aberrations. 18 Furthermore, low PSMA expression caused by tumor heterogeneity 32 might be responsible for occasional false-negative PET/CT results.

It has been proven that ^68^ Ga-PSMA-11 PET/CT reveals the highest contrast in LN metastases, followed by bone metastases, local relapses, and soft tissue metastases. 12 Due to low background signal, ^68^ Ga-PSMA-11 PET/CT allows the detection of bone and organ metastases, 17 which may lead to systemic therapy, but if excluded, may lead to curative therapy. 33

A total of 131 lesions were detected in 20 patients using ^68^ Ga-PSMA-PET/CT, and 123 lesions were detected in 21 patients using ^18^ F-AlF-PSMA-11 PET/CT. Our results corresponded with those reported in the literature. 31 In concordant lesions, there is a strong correlation between the values of ^68^ Ga-PSMA and ^18^ F-AlF-PSMA-11 SUVmax ( *r* = 0.9; *p* = 0.001).

The mean SUVmax in the concordant PSMA-positive lesions was 11.9 for ^68^ Ga-PSMA-11 and 10.5 for ^18^ F-AlF-PSMA-11 ( *p* = 0.001; *n* = 177 lesions). We also found a significantly different median LBR for both tracers: 13.3 (0.95–176) and 12.0 (2.67–132) for ^68^ Ga-PSMA-11 and ^18^ F-AlF-PSMA-11, respectively ( *p* = 0.001).

^68^ Ga-PSMA-11 demonstrated a slightly higher SUVmax, tumor-to-background ratio, and median and mean values as compared with ^18^ F-ALF-PSMA-11 in coincident lesions. These data are also similar to previous reports concerning these PSMA ligands.

Dietlein et al 7 did not find significant differences in SUVmax and tumor-to-background ratios between ( ^18^ F)DCFPyL and ( ^68^ Ga)Ga-PSMA-HBED-CC. However, additional skeletal metastases were observed for ( ^18^ F)DCFPyL as compared with ( ^68^ Ga)Ga-PSMA-HBED-CC. Despite these differences, good correspondence between both tracers was observed. In our study, the ^18^ F-AlF-PSMA-11 SUVmax and LBR were higher in bone-concordant foci compared with ^68^ Ga-PSMA-11 (7.2 0 vs. 8.92 and 14.4 vs. 13.1, respectively). However, a significant time-dependent degradation of [ ^18^ F]AlF-PSMA-11 has been reported and PET acquisition should take place no later than 1 hour postinjection. The limited instability and consequently physiological nonspecific bone uptake due to the potential release of free fluoride might influence SUVmax and LBR calculations, or eventually hamper the visualization of small PCa bone metastases. 25 26

A strong correlation was found between the SUVmax values of gallium and fluorine ( *r* = 0.83; *p* = 0.001) and between the number of lesions detected by both radiopharmaceuticals ( *r* = 0.96). Although there is a very high correlation, we clarify that we cannot conclude identical detection capabilities, since subtle differences in lesion characterization (e.g., intensity, size) could be missed by one tracer over the other.

Hoffman et al found a tendency toward increasing SUVmax with rising PSA. 34 Moreover, Afshar-Oromieh et al described a strong association between PSA level and positive ^68^ Ga-PSMA-11 PET/CT scans. 12 Koerber et al observed a significantly higher mean SUVmax in tumors with higher D'Amico risk classification and Gleason score from biopsy ( *p* = 0.001 for grouped analyses). 35 However, in our research, rising SUVmax values were not associated with rising Gleason categories, PSA levels, or the type of injury detected. These contradictions can be explained by differences in the selected population or characteristics of the research. Hoffmann et al studied several patients with suspected (but not confirmed) prostate carcinoma and he found a “tendency,” but not significant correlation between variables. 34 Afshar-Oromieh et al's results concern the scenario of recurrent PCa, but not initial staging. 12 Koerber et al 35 obtained statistical significance in high-risk but not intermediate-risk tumors.

There is controversy in the literature concerning distant metastasis and regional LN involvement. Based on the results of Steuber et al's research, choline-derivative PET/CT scans did not prove to be useful for LN staging in localized PCa prior to treatment and should not be applied if clinically occult metastatic disease is suspected, suggesting the research of new tracers with higher affinity. 36

As normal lymphatic or retroperitoneal fatty tissue does not exhibit PSMA expression, a metastatic LN can be detected with a favorable lesion-to-background ratio. 31 Initial studies in recurrent PCa scenarios with ^68^ Ga-PSMA described the first promising results. 34 At initial staging, some authors achieved a sensitivity of 88.1% for LN detection with ^68^ Ga-PSMA-PET/CT. 12

Maurer et al suggest that preoperative LN staging with ^68^ Ga-PSMA-PET is superior to standard routine imaging. 31 They were the first to study a large cohort of 130 patients with intermediate-/high-risk PCa, and in a patient-based and template-based analysis, they observed a sensitivity of 65.9 and 68.3%, respectively, with a specificity of 98.9 and 99.1%, respectively, for LN staging.

Many published studies do not recommend routine clinical use of PET/CT technology to detect occult LN metastasis prior to initial treatment because they suggest that detection of microscopical tumor metastases might be missed due to the poor sensitivity of the technique. 36

Giesel and his team demonstrated an excellent sensitivity of 94.7% with ^18^ F-PSMA-1007 PET/CT in detecting LN metastases in the pelvis, including nodes as small as 1 mm, 9 although in other series, the sensitivity to these very small nodes was limited. On the other hand, an analysis evaluating ^68^ Ga-PSMA-PET for LN staging in 30 high-risk PCa patients reported a low sensitivity of 33% and a specificity of 100%. 37 Concerning the excellent sensitivity of ^18^ F-PSMA-1007 (94.7%), 9 versus the low sensitivity of ^68^ Ga-PSMA-PET (33.0%) 37 for LN staging, the mentioned study suffered from several limitations, including a long interval between imaging and surgery or the lack of standardization in regard to imaging protocols and documentation of findings. Therefore, it cannot be concluded that the higher sensitivity in the first cohort is caused solely by the improved tracer. Nevertheless, LN metastases with median diameters of 5 mm are close to the technical resolution limits of PET with ^68^ Ga-PSMA tracers and, therefore, it would be reasonable that ^18^ F-PSMA tracers be perform at a higher level.

Also, concerning the two different sensitivity values for ^68^ Ga-PSMA-PET in LN staging (65.9% for Maurer et al 31 and 33.0% for Budäus et al 37 ), the reasons for this finding can only be speculated as has been highlighted in a recent reply by the authors: a highly dedicated but nonroutine pathological workup with PSMA immunohistochemistry of all resected LN has been performed. However, and probably more relevant, a central standardized review of the PSMA-PET scans performed at external institutions by an experienced nuclear medicine physician was completely missing.

In our study, for the detection of systemic metastasis on a per-patient basis, the sensitivity was the same for both techniques: 0.90 (95% CI: 0.68–0.98). We calculated specificities of 0.96 (95% CI: 0.85–0.99) and 0.94 (95% CI: 0.82–0.98) for ^68^ Ga-PSMA-11 and ^18^ F-AlF-PSMA-11, respectively.

Three LNs in two different patients were histologically positive for metastasis, indicating a false-negative PET/CT result. This is in line with the findings of Budäus et al, who concluded that ^68^ Ga-PSMA-PET/CT performance may be susceptible to the micro-size of tumor deposits (<5 mm). 37

In our initial selection, five patients were excluded because they had additional tumors besides prostate tumors. PSMA expression has been reported in the neovasculature of some solid tumors, such as those related to colon, breast, bladder, and renal cancer, and this fact can limit the technique's specificity. 38

Six false-positive lesions found were in the bone area (2/6 detected by ^68^ Ga-PSMA-11 and 6/6 for ^18^ F-AlF-PSMA-11; Table 2 *). One of them corresponded to a rib pitfall and the others were verified retrospectively by CT as trauma/fracture. In all cases, patients underwent the intended curative primary prostate treatment, and the PSA turned indetectable, confirming the false-positive result. Recent studies have reported occasionally increased radiolabeled PSMA uptake in rib fractures, as well as in benign bone diseases, such as fibrous dysplasia and Paget's disease. 39 As such findings must be taken into account when reporting PET/CT results, the presence of benign disease was an exclusion criterion for our clinical assay.

Although the literature refers to false-positive findings in relation to uptake in retroperitoneal celiac ganglia, 40 in this analysis, they were easily identified and not taken into account.

Concerning ethical limitations, positive PET/CT extra-pelvic findings were not histopathologically validated in most patients, as many studies reported in the literature. 35 However, it is not possible to assess all lesions in multimetastatic patients, and on the other hand, small lesions are difficult to reach anatomically. The high sensitivity in this research may be related to the relatively low average follow-up time, which may have prevented distant metastases from becoming clinically evident in our time window. Obviously, it is important to note that our study focuses on patient-based diagnostic values. Finally, although our study has more patients than other published studies, we highlight the need for prospective studies with adequate numbers of patients to achieve strong statistical significance.

Both PET/CT studies accordingly changed the management in 20/66 patients (30%) from supposedly curative therapy options (radiotherapy/prostatectomy) to systematic therapy in cases of distant metastasis, radically changing patients' management.

## Conclusion

^68^ Ga-PSMA-11 and ^18^ F-AlF-PSMA-11 PET/CT seem to be clinically equivalent imaging techniques for the assessment of primary intermediate-/high-risk PCa with great potential for the detection of metastatic spread that would impact patient management.

Further prospective studies with larger cohorts are needed to fully include PSMA-PET/CT in clinical practice guidelines for the assessment of patients with primary intermediate-/high-risk PCa.

**Table 2:** Patients at initial staging

	^18^ F-AlF-PSMA					^68^ Ga-PSMA			
Patient	Bone	Lymph node	Others	Total	Bone	Lymph node	Others	Total
2	3/3	1/1	1/1	5/5	1/3	1/1	1/1	3/5
3	1/1 a	Negative	Negative	1/1	1/1 a	Negative	Negative	1/1
4	6/6	1/1	Negative	7/7	5/6	1/1	Negative	6/7
5	2/2	3/5	Negative	5/7	1/2	5/5	Negative	6/7
7	4/4 a	Negative	Negative	4/4	0/4	Negative	Negative	0/4
18	15/15	8/10	Negative	23/25	11/15	10/10	Negative	21/25
19	1/4	Negative	Negative	1/4	4/4	Negative	Negative	4/4
26	4/4	5/5	Negative	9/9	3/4	5/5	Negative	8/9
27	Negative	2/4	Negative	2/4	Negative	4/4	Negative	4/4
28	5/5	10/10	Negative	15/15	5/5	10/10	Negative	15/15
31	Negative	2/5	Negative	2/5	Negative	5/5	Negative	5/5
35	3/3	6/6	Negative	9/9	3/3	6/6	Negative	9/9
40	Negative	4/5	Negative	4/5	Negative	5/5	Negative	5/5
46	2/2	Negative	Negative	2/2	2/2	Negative	Negative	2/2
48	3/3	2/2	Negative	5/5	2/3	2/2	Negative	4/5
50	Negative	6/6	Negative	6/6	Negative	6/6	Negative	6/6
55	Negative	10/10	Negative	10/10	Negative	10/10	Negative	10/10
57	Negative	7/13	Negative	7/13	Negative	13/13	Negative	13/13
59	1/1	2/5	Negative	3/6	1/1	5/5	Negative	6/6
61	2/2	Negative	Negative	2/2	2/2	Negative	Negative	2/2
65	1/1 a	Negative	Negative	1/1	1/1 a	Negative	Negative	1/1
Total	53/56	69/88	1/1	123/145	42/56	88/88	1/1	131/145

Note: Positron emission tomography (PET)/computed tomography (CT) extra-prostatic lesion localization. Patients with both negative ^18^ F-AlF-PSMA and ^68^ Ga-PSMA are not shown.

a False-positve results in bone lesions.
